# Experimental radar data for monitoring brain atrophy progression

**DOI:** 10.1016/j.dib.2022.108379

**Published:** 2022-06-10

**Authors:** Rahmat Ullah, Imran Saied, Tughrul Arslan

**Affiliations:** School of Engineering, University of Edinburgh

**Keywords:** Alzheimer's disease, Microwave imaging data, Brain atrophy, Brain imaging, Radar data

## Abstract

This data set contains complex frequency domain signals obtained from unidirectional antennas mainly fabricated for radar-based head imaging. Data were obtained as part of a project investigating radar-based microwave imaging for monitoring neurodegenerative diseases, especially Alzheimer's disease. The wearable device, measurement setup, and phantoms used are described. Multiple experiments were performed to get the data from three lamb brain phantoms that realistically mimic the whole-brain atrophy due to Alzheimer's disease. Microwave imaging has shown great potential for breast and brain screening due to its low cost, non-ionizing, portable, and wearable nature. Most of the studies are based on simulations with good results, but further evaluation on experimental data is required before its clinical viability. This work provides an open-source experimental dataset that can be used to evaluate novel signal processing and imaging techniques and validate simulation results. The data provide both the magnitude and phase value at each discrete frequency, making this data set useful for both time-delay and phase-shift based imaging algorithms.

**Specifications Table**

**Table:** 

Subject	Biomedical Engineering, Medical imaging
Specific subject area	Electromagnetic Imaging
Type of data	Table (Microsoft Excel Worksheet (.xlsx))
How the data were acquired	Data were obtained from experiments on lamb brain phantoms moulded into a skull model, using six unidirectional fully-textile ultrawideband antennas mounted into a flexible hat structure. The signals are generated with an HP 8753 Vector Network Analyzer (VNA) with a frequency range of 0 to 3 GHz.
Data format	Raw (Labelled)
Description of data collection	The data set contains complex signals obtained using six antennas from phantoms mimicking three different levels of brain atrophy. Ten lamb brains were frozen and inserted into the skull cavity to mimic a typical brain. A determined portion of the lamb brain was removed equally throughout the outer layer to mimic different cases of whole-brain atrophy. The outer layer was accumulated using a phantom representing Cerebrospinal Fluid (CSF). Wearable and portable wearable device consisting of six flexible antennas were used to obtain the data. The personal computer was connected to the VNA to send a signal transmission command. For this purpose, the GPIB connector with NI488.2 API, which includes software to command the VNA, is utilized. Specifically, the VNA Utility software that is part of the GPIB toolkit developed from KE5FX for GPIB controlled equipment is used. This software allows to capture and save measurements from the VNA onto a PC [1]. The scattered signals were recorded at the same port for each antenna by turning on the switch connected with the active element and turning off other switches. The data were saved for different pairs of antennas. The same antenna pair were used for all three sets of experiments.
Data source location	• Institution: School of Engineering, The University of Edinburgh • City/Town/Region: Edinburgh, United Kingdom
Data accessibility	Repository: Experimental radar data for monitoring brain-atrophy progression URL: https://data.mendeley.com/datasets/y8syxphvnr/1 Doi: 10.17632/y8syxphvnr.1
Related research article	Ullah, Rahmat, Imran Saied, and Tughrul Arslan. " Measurement of whole-brain atrophy progression using microwave signal analysis." Biomedical Signal Processing and Control 71 (2022): 103083. https://doi.org/10.1016/j.bspc.2021.103083

**Value of the Data**

• The data can be used to improve the existing signal processing and imaging techniques for microwave-based head diagnostics, especially for detecting and monitoring neurodegenerative diseases such as Alzheimer's disease.
• The researchers working on radar imaging or signal processing can significantly benefit from this experimental data, as most studies rely on simulation due to the scarcity of experimental data.
• These data can be used to investigate and validate newly proposed signal processing or imaging techniques for detecting or monitoring neurodegenerative disease.
• The data can be used in both time-delay and phase-shift algorithms for head imaging. The
• frequency-domain signals can be converted to the time domain using transformation algorithms such as Fourier transforms.
• The dataset provides both the magnitude and phase information for each antenna that can be used to investigate artefact removal techniques to eliminate the clutter effects, for example, skull reflections or antenna couplings during signal preprocessing [2].

## 1 Data Description

Based on the reflection and transmission coefficient measurements, the dataset provides the recorded radio frequency (RF) data for physiological changes (whole-brain atrophy) in the human brain due to Alzheimer's disease. The reflection coefficient (S_11_) is the difference between the amplitudes of the reflected and incident waves. The transmission coefficient (S_21_) is the ratio of the transmitted wave's amplitude to that of the incident wave. The data has been used in our recent study that investigates the use of radar-based imaging for detecting and monitoring neurodegenerative disease detection [3]. The data set contains a collection of all S-parameter measurements with the .xlsx extension. The data files in this folder contain the frequency-domain S-parameters before any reference-scan subtraction has been performed. Two-port VNA is used where two antennas operate at one time. Each excel sheet contains data for two antennas. The data include the frequency (GHz), magnitude, and Phase (Degree) for S_11_, S_21_, S_12_, and S_22_. The sheet's name reflects which antenna combinations are used. For example, the first sheet," Normal1and4", means Normal (healthy) brain phantom, and antennae one and four are used. There are a total of seven antenna combinations for each set of experiments.

**Fig. 1:**
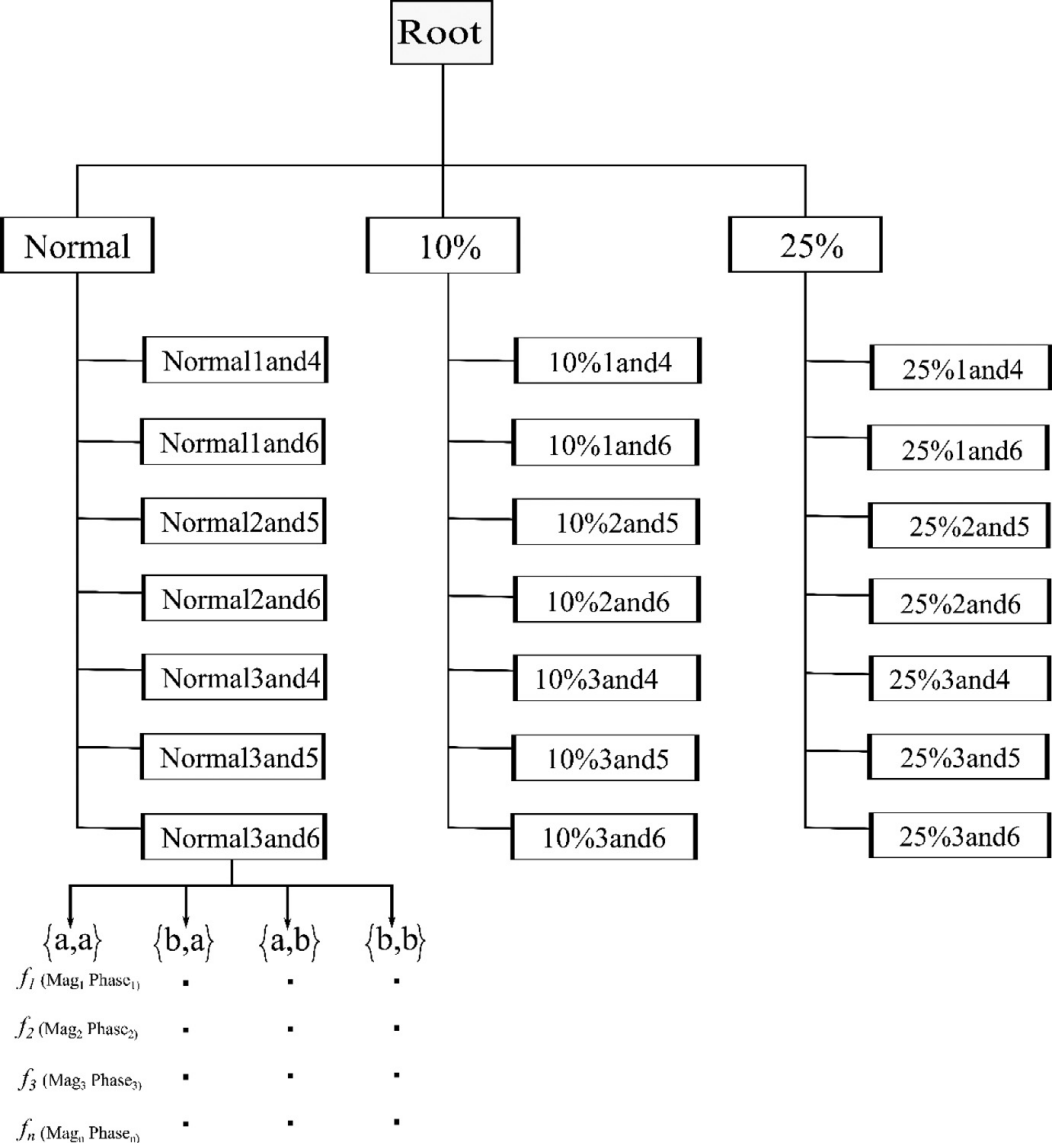
The Data structure of the repository. There are three categories of data corresponding to phantoms used for experimenters. Each class has seven sheets for each pair of antenna.

Fig. 1 shows the structure of the data repository. There are three categories i.e., Normal, 10% atrophy, and 25% atrophy. Each of these categories contains data for different pairs of antennas. The sheet name reflects which antenna pairs are used. For example, the sheet name" Normal1and4" means, Normal (healthy) phantom and the combination of antenna one and four.

Similarly," 10%1and4" means 10% (mild) atrophy phantom and the combination of antenna one and four. There are seven antenna combinations for each phantom that covers the whole head. The exact combinations of antennas are used for the other two sets of experiments.

Each sheet contains a column for the frequency range from 0.2 to 3 GHz. The total number of discrete frequency points for each antenna is 201. Each antenna combination contains both reflected and transmitted signals at the corresponding frequency. These are denoted as S{a,a}, S{b,a}, S{a,b} and S{b,b} in Fig. 1, where a and b denotes the first and second antenna respectively, in the antenna pair used. Each reflected and transmitted signal has a magnitude (dB) and phase (degree) value at the corresponding frequency. The data format is similar for all three categories and antenna pairs' combinations.

The data can be transformed to a time-domain using transformation algorithms such as IFFT [4]. It can be loaded using MATLAB, Python, or any other language. By default, the value for magnitude and phase are stored in separate columns; however, these can be merged to make it a complex data type.

## 2 Experimental Design, Materials and Methods

**Fig. 2:**
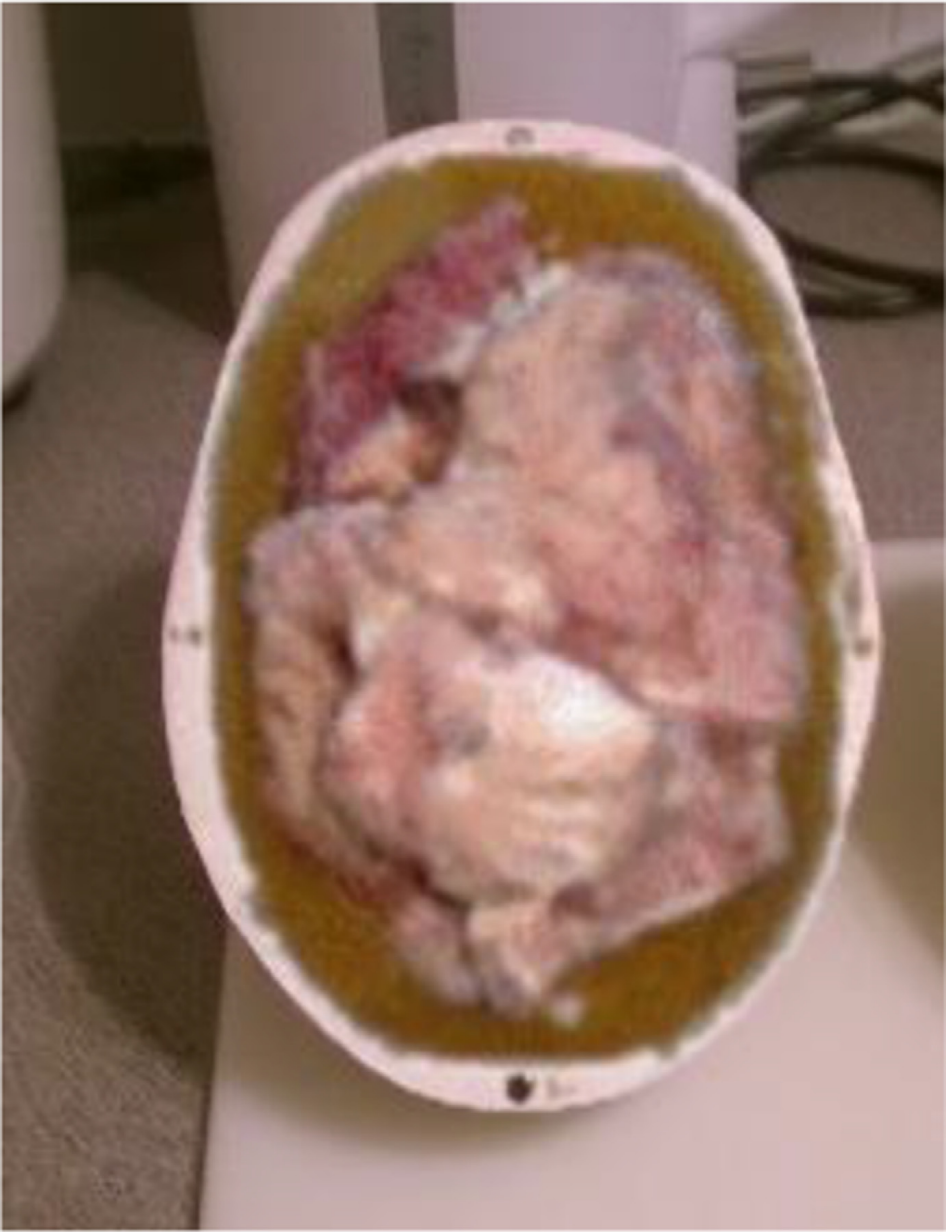
Lamb brain b) 10% brain atrophy (mild case).

A wearable device with six antennas placed at an equal distance was used for experiments [5]. Experiments were performed on 3 cases 1) Healthy (0% brain atrophy), 2) Mild atrophy (10%), and 3) Severe atrophy (25%). Fig. 2 shows a lamb brain phantom used to represent mild atrophy. More Details about phantoms for all atrophy cases can be found in our previous work [3].

**Fig. 3:**
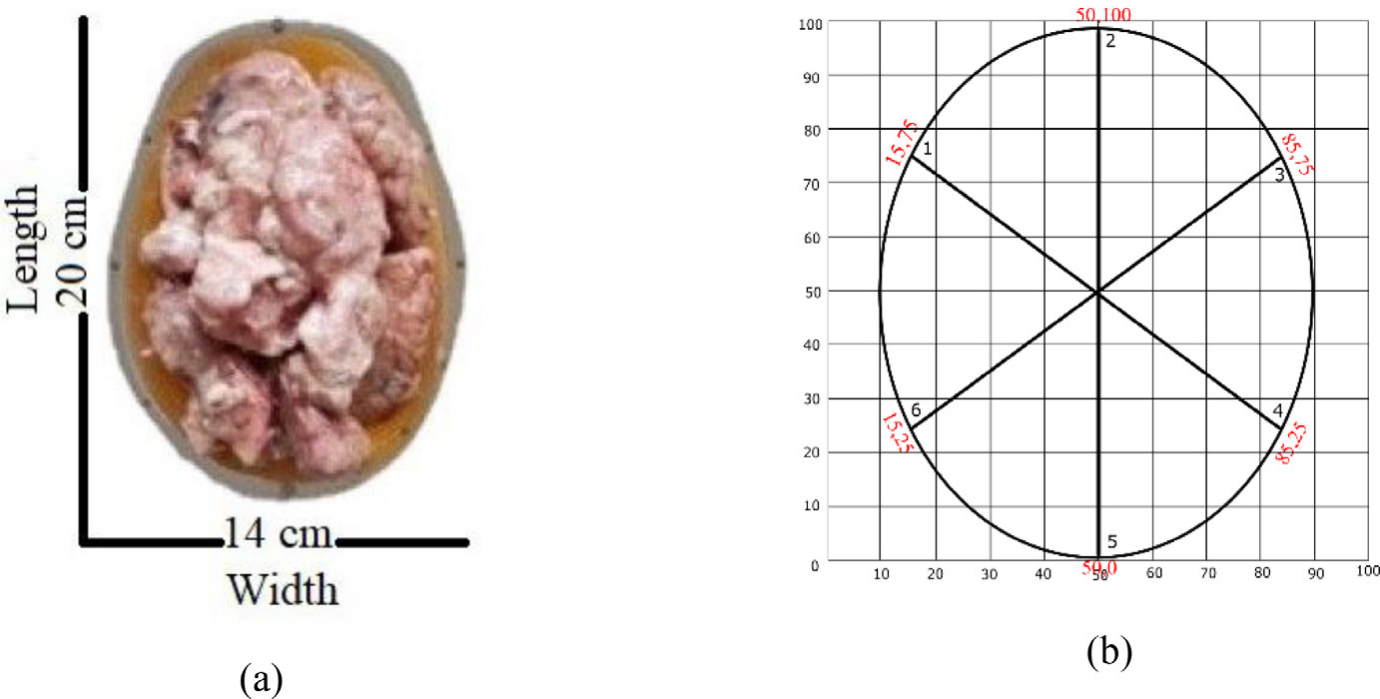
Lamb brain Phantoms used to mimic the whole-brain atrophy moulded into a skull model of 20 × 14 × 18 (length x Width x Height). (b) Antenna locations in Cartesian Coordinates based on their placement in the wearable device.

The imaging setup can either be monostatic or multistatic, with one or more antennas acting as transmitters and receivers [6]. A single antenna is used to transmit and receive the signals in a monostatic configuration. In this work, a pair of antennas are used, where both antennas act as transmitter and receiver, one active at a time. Each antenna in the antenna pair transmitted the signal, and reflected and transmitted signals were captured by both antennas. The experiments were repeated for multiple pairs of antennas and both the reflected and transmitted signals were stored.

Six flexible ultrawideband (UWB) antennas have been fabricated and used in wearable device. These antennas were placed at equal distances around the phantom. The antennas were made with Shieldex Zell, a 0.1-mm-thick flexible textile, as a conductive material and RS-PRO Viscose Wool Felt as a substrate. The loss tangent was determined to be 0.068, and the substrate's relative permittivity was found to be 1.55. The length and width of the antenna measure 85 and 35 mm prospectively. The outer layer is a 19 mm thick dielectric absorber used to ensure that backward radiation emitted by the antenna is blocked. The absorber is a conformal absorbing material backed with a conductive sheet that is typically used to suppress the back lobe radiation of monopole antennas. For our experiments, the absorbing material that was used is a flexible dielectric absorber, AN70 from Emerson & Cuming, covered with flexible conductive textile that not only suppresses the back lobe radiation of the probing antennas but also acts as a mount and hat-like frame to hold the antennas sensors. The antennas and absorber were separated with a 5 mm thick foam to achieve good penetration. To maintain the integrity of antennas, the measured dielectric value of the foam is equal to that of air. Further information about the antenna can be found in [7]. The experiments were repeated for each antenna to collect both reflected and transmitted signals (S_11_ and S_21_, respectively). For each antenna, a total of 201 frequency points were saved.

A normal lamb brain size is about 1/10th of a healthy human brain. Therefore, ten frozen lamb brains were put into the skull cavity to mimic a normal brain. A life-size skull model was used containing similar indentations that are present in human beings. In addition, the skull model could be disassembled easily, allowing contents to be placed inside. A typical brain's structure has a volume of 1200 cm3. To mimic different cases of whole-brain atrophy, a measured amount of brain was removed throughout the outer layer, and the empty area was filled with CSF. For example, around 10% of the lamb brain was removed equally across the outer layer of the phantom for mild atrophy. Agar and saline water were combined to create this artificial phantom representing CSF. A colour dye was used to make the CSF layer visible. In terms of permittivity and conductivity, multiple measurements were performed to check if they were in the same range as the actual CSF. The phantoms were measured to be within 2% of the real dielectric properties of CSF.

Similarly, a quarter of a lamb's brain was removed to mimic severe atrophy (25%). In this case, the vacant region was refilled with a thick layer of artificial CSF phantom. The image (Top view) of a lamb brain moulded into a skull model is shown in Fig. 2 (a). The hat-shaped wearable device shown in Fig. 2 (b) has six fabricated antennas covering the entire head placed at equal distances.

Experiments with complete (healthy), 10% (for mild), and 25% (for severe) atrophy brain phantoms were used to generate the signal data. The brain tissues were put into the skull model, and the gaps were filled with artificial CSF tissue. In practice, the area after brain atrophy is accumulated with CSF. It also forced to remove any air in between tissues and the tissues and the skull model. The wearable device having six antennas mounted on the inner side of the absorber was used. These antennas are fully-textile directional antennas with wide-band capabilities. The distance between the antennae was 9 mm. The antenna locations in Cartesian coordinates can be found in Fig. 2(b). These locations are essential for both frequency and time-domain imaging algorithms. A vector network analyzer (VNA) generated and received the signals. Multiple experiments were performed with healthy brain size and a decrease of 10% and 25%, respectively. The generated signals were stored on a PC using VNA Utility software, part of the GPIB toolkit [1].

## Ethics Statements

The lamb brain samples used in the experiments were obtained from a local butcher. The study does not involve in vivo experiments on animals. Therefore, no permissions were required.

## CRediT Author Statement

**Rahmat Ullah:** Conceptualization, Methodology, Data curation, Software, Writing – original draft, Writing – review & editing, Formal analysis; **Imran Saied:** Data curation, Writing – review, & editing, Formal analysis; **Tughrul Arslan:** Formal analysis, Supervision, Project administration.

## Funding

This research did not receive any specific grant from funding agencies in the public, commercial, or not-for-profit sectors.

## Declaration of Competing Interest

The authors declare that they have no known competing financial interests or personal relationships that could have appeared to influence the work reported in this paper.

## Data Availability

Experimental radar data for monitoring brain-atrophy progression (Original data) (Mendeley Data).
